# Complete mitochondrial genome of the freshwater snail *Tarebia granifera* (Lamarck, 1816) (Gastropoda: Cerithioidea: Thiaridae)

**DOI:** 10.1080/23802359.2022.2026832

**Published:** 2022-01-24

**Authors:** Nan Yin, Shuai Zhao, Xiao-Chen Huang, Shan Ouyang, Xiao-Ping Wu

**Affiliations:** School of Life Sciences, Nanchang University, Nanchang, China

**Keywords:** Mitogenome, phylogeny, cerithioidean, parasitic disease

## Abstract

The freshwater gastropod *Tarebia granifera* (Lamarck, 1816) is found in Taiwan, Hainan, and Guangdong provinces in China, and is one of the main intermediate hosts of trematodes that infect humans. The taxonomic positions of some cerithioidean families are still unclear, and whole mitochondrial genome studies are scarce in the Thiaridae. In this study, we describe the complete mitogenome of *Tarebia granifera* (Lamarck, 1816). The mitogenome is 15,555 bp in length, with a total of 37 genes, including 13 protein-coding genes, 2 rRNA genes, and 22 tRNA genes. It is consistent with the essential features of previously studied mitochondrial genomes of species belonging to the superfamily Cerithioidea. Our study demonstrates the usefulness of mitogenomic data for resolving phylogenetic relationships of families within Cerithioidea and may also contribute to the prevention and control of the parasitic diseases caused by trematodes, which use *T. granifera* as an intermediate host.

The superfamily Cerithioidea currently includes 21 families (Bouchet et al. 2017; Neiber and Glaubrecht 2019d), nine of which inhabit freshwater environments: Amphimelaniidae, Hemisinidae, Melanopsidae, Pachychilidae, Paludomidae, Pleuroceridae, Semisulcospiridae, Thiaridae, and Zemelanopsidae (Campbell 2019; Glaubrecht and Neiber 2019a, 2019b; Neiber and Glaubrecht 2019a, 2019b, 2019c, 2019d; Strong and Lydeard 2019). Initially, most of the freshwater species in the superfamily Cerithioidea were placed in the family Melaniidae and the genus *Melania* (Neiber and Glaubrecht 2019b). With the development of molecular systematics, it was found that the cerithioidean freshwater taxa were not monophyletic, and the taxonomic positions of some cerithioidean families are still unclear (Strong et al. 2011; Neiber and Glaubrecht 2019d). *Tarebia granifera* (Lamarck, 1816) is an invasive species native to South and Southeast Asia and several Western Pacific Islands. In addition, the species has become widely invasive in the tropics outside its native range (e.g. Africa, the Mediterranean region and the Middle East, as well as North, Central and South America), with the spreading being attributed to the aquarium trade or dispersal by birds (Malatji et al. 2021; Chalkowski et al. 2021). In China, *T. granifera* is mainly distributed in the provinces of Taiwan, Hainan, and Guangdong (Liu et al. 1993). *Tarebia granifera* is one of the main intermediate hosts of *Paragonimus westermani* and *Metagomimus yokogawai* (Liu et al. 1993; Veeravechsukij et al. 2018a, 2018b). The complete mitochondrial genome of this species is still lacking. By sequencing the mitochondrial genome of *T. granifera*, our study provides additional information on mitogenome evolution and the phylogeny of Cerithioidea, and may also contribute to the prevention and control of the parasitic disease caused by trematodes, which use *T. granifera* as an intermediate host.

Specimens of *Tarebia granifera* were collected from Pingshan Wetland Park (22°41′53″N, 114°22′4″E), Shenzhen, China, in 2019. Morphological identification followed the literature (Liu et al. 1993) and specimens from the Institute of Zoology, Chinese Academy of Sciences. Tissues were preserved at −80 °C in a refrigerator, and the voucher specimen (number: 21-NCU-XPWU-TG01; contact Xiao-Ping Wu: xpwu@ncu.edu.cn) was deposited in the Museum of Biology in Nanchang University. Referring to the previous study, the mitogenome of *Tarebia granifera* was sequenced using primer-walking (Xie et al. 2019). Firstly, *cob* (Merritt et al. 1998), *rrnL* (Palumbi et al. 1991), and *cox1* (Folmer et al. 1994) genes were obtained by universal primers. Secondly, three sets of primers were designed to amplify the complete mitogenome into three long fragments. Details of primers and PCR conditions are shown in Supporting information S1. Finally, sequences were assembled into the whole mitogenome by SeqMan program (DNAstar).

ARWEN (Laslett and Canbäck 2008) and MITOS (Bernt et al. 2013) were used jointly to identify localized transfer RNA (tRNA) genes and analyze secondary structures. Ribosomal RNAs (12S rRNA and 16S rRNA) were then compared by homology with other Cerithioidea species. Open Reading Frame Finder (ORF Finder) (http://www.ncbi.nlm.nih.gov/orffinder/) and BLAST searches were used to identify 13 protein-coding genes. We downloaded 12 published gastropod mitogenomes from GenBank, and used *Tricula hortensis* (Truncatelloidea: Pomatiopsidae) as outgroup. Phylogenetic analysis was conducted for Bayesian inference (BI) in MrBayes (Ronquist et al. 2012) based on the best-fit partitioning schemes and models (details in Table S2) selected by PartitionFinder (Lanfear et al. 2017). Two simultaneous runs with four independent chains were implemented for 10 million generations, sampling every 1000 generations. The first 25% of these trees were discarded as burnin.

The mitogenome of *Tarebia granifera* is 15,555 bp in length (GenBank accession number: MZ662113). It contains 37 genes that encode two rRNAs, 22 tRNAs, and 13 proteins. The mitogenome base composition was A (30.9%), T (34.5%), C (17.7%), and G (16.9%), with the A＋T content of 65.4% being remarkably higher than the G＋C content (34.6%), which was similar to other Cerithioidea (Lee et al. 2019). The total length of protein-coding genes is 11,226 bp, accounting for 72.17% of the complete mitochondrial genome. The 12S rRNA (958 bp) gene was located between the *trnS* and *trnT* genes and the 16S rRNA (1,354 bp) gene was located between the *trnV* and *trnL* genes, respectively. This mitogenome contained 22 tRNA genes, with the shortest tRNA genes *trnY*, *trnH* and *trnM* being 65 bp in length, and the longest tRNA genes *trnW*, *trnA* and *trnE* being 70 bp in length.

The tree topology obtained in this study is consistent with previous studies (Wang et al. 2017; Lee et al. 2019). Phylogenetic analysis based on 12 PCGs and two rRNA genes showed that *Tarebia granifera* and *Pseudocleopatra dartevellei* had a close relationship (Bayesian posterior probability = 1), forming the sister cluster of *Cerithidea sinensis* and *Cerithidea obtusa* (Figure 1). The majority of the tree nodes were well-resolved with high posterior probabilities. Our results demonstrate the usefulness of complete mitochondrial genome in resolving phylogenetic relationships among cerithioidean lineages.

**Figure 1.**
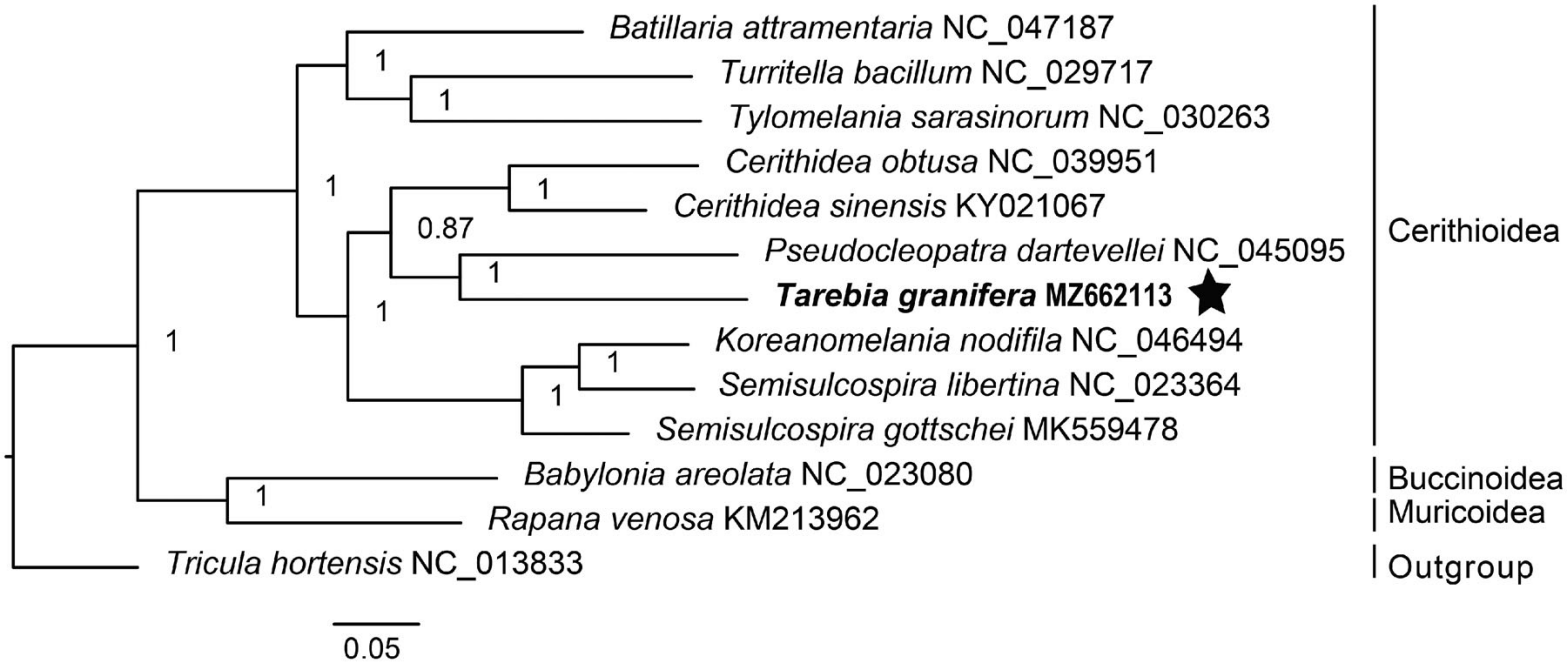
Bayesian 50% majority-rule consensus tree of 10 cerithioidean gastropods based on 12 mitochondrial PCGs and two rRNA genes. The analysis included also representatives of the superfamilies Buccinoidea and Muricoidea. *Tricula hortensis* (Truncatelloidea) was used as outgroup.

Additional supporting information may be found online in the Supporting information section at the end of the article.

## Ethical approval

The handling of freshwater snails was conducted in accordance with the guidelines on the care and use of animals for scientific purposes set by the Institutional Animal Care and Use Committee (IACUC) of Nanchang University, Jiangxi, China.

## Data Availability

The genome sequence data that support the findings of this study are openly available in GenBank of NCBI at (https://www.ncbi.nlm.nih.gov/) under the accession no. MZ662113.
